# Structure Optimization of Cellulose Nanofibers/Poly(Lactic Acid) Composites by the Sizing of AKD

**DOI:** 10.3390/polym13234119

**Published:** 2021-11-26

**Authors:** Lei Li, Minjian Cao, Jingdan Li, Cong Wang, Shengjuan Li

**Affiliations:** School of Materials Science and Engineering, University of Shanghai for Science and Technology, 516 Jungong Road, Shanghai 200093, China; 18639819198@163.com (M.C.); ljd980414@163.com (J.L.); wangcong_0311@163.com (C.W.); usstshenli@usst.edu.cn (S.L.)

**Keywords:** cellulose nanofibers, alkyl ketene dimer, poly(lactic acid), surface treatment, sizing, green composite, mechanical properties

## Abstract

Recently, cellulose nanofibers (CNF) are used as one novel fillers to reinforce poly(lactic acid) (PLA) matrix and form PLA green nanocomposites. In the present work, alkyl ketene dimer (AKD) was used as the sizing of CNF to improve the interfacial compatibility between the hydrophilic CNF and the hydrophobic PLA. The interactions between the AKD and CNF were characterized by X-ray diffraction (XRD) and Fourier transform infrared spectroscopy (FTIR), which showed the formation of ketone ester structure between AKD and the hydroxyl groups of CNF. Thermo gravimetric analysis (TGA) showed the little reduced thermal stability of the AKD-CNF/PLA composites. The AKD-CNF/PLA morphology has rough surfaces due to the incorporation of cellulose nanofibers. The mechanical properties of AKD-CNF/PLA were tested by tensile testing, which discovered more AKD-CNF content enhances stress–strain performance. The highest tensile strength of composites was obtained for PLA with 5.0 wt.% AKD-cellulose, which is almost nine times higher than that of the pure PLA.

## 1. Introduction

Currently, the plastic industry encounters two critical problems. One is the depletion of energy resources in respect of the conventional petroleum-based materials. The other one is the environmental pollution caused by such petroleum-based waste products [1,2]. In order to decrease the over exploitation of nonrenewable resources, material scientists and engineers are giving top priority to developing biodegradable and recyclable biopolymers [3]. As one important biopolymers, poly(lactic acid) (PLA) can be made from renewable biomass such as lignocellulose, corn, beet sugar and so on and is fully biodegradable [4,5,6,7,8]. PLA is an thermal plastic aliphatic polyester, which has a chiral center in the structure to form optical active substance of PLA, including poly(L-lactic acid) (PLLA), poly(D-lactic acid) (PDLA) and poly(L,D-lactic acid) (PDLLA) [9]. From production to after utilization, the carbons in PLA originate from carbon dioxide in air and do not discharge spare carbon dioxide into atmosphere [10]. With good mechanical properties to overtake traditional plastics, PLA was expected to substitute some of the nondegradable plastics for applications in the packaging [11], electronics [12], automotive [13] and biomedical fields [14]. Nevertheless, PLA is brittle and apt to deform when it is heated, leading to inferior to many petroleum-based polymers and limited applications of pure PLA [15,16,17]. In order to fully exploit PLA potential, the chemical, physical and mechanical properties of PLA is desirable to be improved greatly. One promising approach is to prepare nanocomposites of PLA with reinforcing elements. It is well known that solvent casting is the most popular and simplest technique to fabricate nanocomposites [18]. In the solvent casting process, dissolved or dispersive materials are poured on a flat substrate and subsequently form nanocomposites when the solvent is evaporated under suitable humidity, temperature and pressure. PLA can be successfully dissolved in many solvents, such as water [19,20], acetone [21] and dichloroethane [22]. Thereby a series of PLA-based biopolymer/nanofiller nanocomposites were successfully prepared and reported, including carbon nanotubes [23], cellulose nanocrystals [24], nanosilica [25] and carbon dots [26]. Cellulose nanofibers (CNF) are among the interesting reinforcing nanomaterials with many advantages, such as good mechanical properties, low density, biodegradability and abundant resources with low cost. CNF attracts intensive interests to develop environmentally friendly practical applications of PLA nanocomposites [26,27,28].

Cellulose is highly hydrophilic due to plenty of hydroxyl groups on the main chain, which are commonly incompatible to the hydrophobic interfaces of polyester. Such discrepancy can hinder the homogeneous dispersion of CNF on PLA matrix and reduce the mechanical properties of the nanocomposites. To cure such discrepancy, many methods have been developed to tailor the interfaces between cellulose and PLA, including adding crosslinking agents [29]. Alkyl ketene dimer (AKD) is one traditional sizing agent in the paper industry [30,31], which is less susceptible to hydrolysis and can offer hydrophobization of cellulose fibers efficiently with a small quantity in the water-based solution [31,32]. To our best knowledge, AKD has not been reported on the surface modification of CNF (AKD-CNF) for PLA composite application.

In this work, a facile method is used to modify the surface of CNF by AKD emulsion and then disperse the modified CNF in the PLA/dimethyl sulfoxide (DMSO) solution to obtain nanocomposite successfully. The effect of addition of AKD-CNF in the PLA matrix is investigated. The structures, morphology, and thermal stability of reinforced PLA by AKD-CNF are characterized. Mechanical properties of PLA composites are also measured in the present work.

## 2. Materials and Methods

### 2.1. Materials

PLA (Mw = 110,000), dimethyl sulfoxide (DMSO) and absolute ethanol were purchased from Beijing Huaweiruike Chemical Co. Ltd. (Beijing, China) Dissolving pulps were kindly supplied by Shandong Yamei Sci-Tech Co. Ltd. (Binzhou, China). AKD emulsion was kindly supplied by Shanghai Dongsheng New Materials Co. Ltd. (Shanghai, China).

### 2.2. Preparation of Nanocomposites

#### 2.2.1. AKD Modification of Cellulose Nanofibers (AKD-CNF)

The pulp was mechanically treated by a high-speed blender at extremely high stirring speed to obtain individualized nanofibers. The AKD emulsion was added into CNF water suspension (1% *v*/*v*) by 0.5:50, 1.5:50 and 2.5:50 (*v*/*v*) to yield final concentrations of 1.0, 3.0, and 5.0% *v/v* of AKD. The suspension was sonicated for 15 m at room temperature. Thereafter, the suspension was put in an oven at 50 °C for 30 min, 70 °C for 20 min or 105 °C for 5 min, respectively, to ensure fully reaction of AKD with CNF. Then the AKD-CNF was ultrasonicated for 30 min and centrifuged. Absolute ethanol was added to the precipitation and ultrasonicated for 15 min, followed by centrifugation. The process was repeated until water was totally replaced by ethanol.

#### 2.2.2. AKD-CNF/PLA Composites Preparation

The desired amount of PLA was dissolved in DMSO at a temperature of 70 °C. Then various amount of AKD-CNF was added into the PLA-DMSO solution and magnetically stirred to form a homogeneous suspension. The mass ratio of AKD-CNF/PLA is set to 1.0, 2.5 and 5 wt.%. Then, the suspension was ultrasonicated for 15 min and then poured onto a glass pan. The solvent evaporated to form film composites. The composites were cured at 50 °C in an electric heating air-blowing drier to ensure the completely removal of DMSO.

### 2.3. Characterization

The X-ray diffraction (XRD) measurements were conducted on the Bruker D8 ADVANCE X-ray diffractometer (Bruker, Karlsruhe, Germany) with Cu kα as the radiation source (λ = 1.5406 Å). The X-ray diffractometer works under a voltage of 40 kV and a current of 40 mA. The samples were scanned in a range of 2θ = 10–50° at a speed of 5.0°/min.

The Fourier transform infrared spectroscopy (FTIR) was used to analyze the interaction between AKD and cellulose nanofibers on a Fourier transform spectrometer (Spectrum 100, Perkin Elmer, Rodgau, Germany) in a wavenumber range of 500–4000 cm^−1^. Fifty scans were accumulated at a resolution of 2 cm^−1^.

The thermal stability of AKD-CNF/PLA was evaluated through thermo gravimetric analysis (TGA). The equipment used in this work was Pyris 1 (Perkin Elmer, Waltham, MA, USA). 10 mg of samples were heated at a rate of 30 °C/min from 30 to 600 °C, under N_2_ flows for both the balance and the sample (20 mL/min) to prevent thermoxidative degradation.

The tensile strength and elongation of the samples were measured using a universal mechanical machine (UTM, 5567A, Instron, Norwood, MA, USA) at a head speed of 2 mm/min. Specimens were hot pressed into a dumbbell shape with a length of 100 mm, a width of 10 mm and 5 mm thick before the mechanical test.

The morphology of AKD-CNF/PLA composite surfaces were analyzed using field emission scanning electron microscopy (SEM, FEI Quanta 450, Hillsboro, OR, USA) at an electron acceleration voltage of 30 kV. All the surfaces were sputtered with gold before the SEM measurement.

## 3. Results

### 3.1. Surface Modificaton of Cellulose Nanofibers

**Figure 1:**
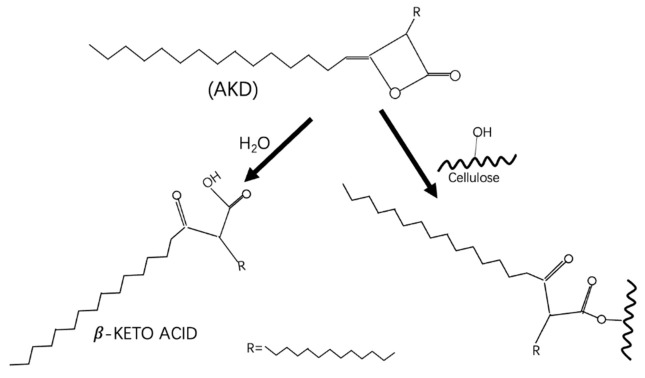
The AKD reaction mechanism on the cellulose surface and in the water.

AKD was obtained from the dimerization of fatty acids, typically ranging C14 to C20. Although nonconsensus was achieved, AKD was proposed to react with hydroxyl groups of cellulose molecules to form a β-keto-ester, or with water form a β-keto-acid (Figure 1) [33].

### 3.2. XRD Characterization

**Figure 2:**
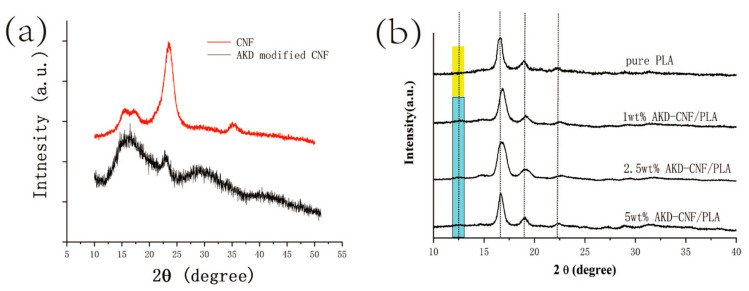
XRD patterns of (**a**) CNF (top) and AKD modified CNF (bottom); (**b**) AKD-CNF/PLA composites.

Figure 2a presents the XRD patterns of CNF and AKD-CNF, respectively. As shown in the figure, the XRD pattern of CNF changed greatly after AKD addition to demonstrate the changes of cellulose crystalline structure and crystallinity. The intensity of the characteristic peak of 2θ = 22° decreased greatly indicates the low crystallinity of AKD-CNF. The reason for such a change is attributed to the high specific surface area of nanofibers from the nanometerization of cellulose. More AKD molecules are adsorbed onto the fiber surfaces, which facilitates the reaction between AKD and cellulose. As a result of the esterification of cellulose by AKD, abundant exposed methyl and methylene groups over the fiber surfaces to result in the hydrophobicity of CNF. Meanwhile, methyl and methylene groups can break the hydrogen bonded structure, lowing the crystallinity of modified CNF.

Figure 2b presents XRD patterns of PLA and AKD-CNF/PLA composites with a series of mass ratios. One new diffraction peak at 2θ = 13° is observed in the profiles of AKD-CNF/PLA, which is the characteristic peak of cellulose as shown in Figure 2a. Meanwhile, the position and shape of the characteristic peaks in the XRD pattern of AKD-CNF/PLA are different from the ones of the pure PLA at 2θ = 16.5, 19 and 22.5°, showing the variation of the PLA crystalline size due to the AKD-CNF interaction. The peaks at 2θ = 19 and 22.5° turn to wider for 1 and 2.5 wt.% AKD-CNF/PLA although the peak at 2θ = 22.5° is narrowed in the 5 wt.% CNF/PLA XRD pattern, showing an optimal dispersion of CNF is less than 5 wt.%.

### 3.3. FTIR Characterization

**Figure 3:**
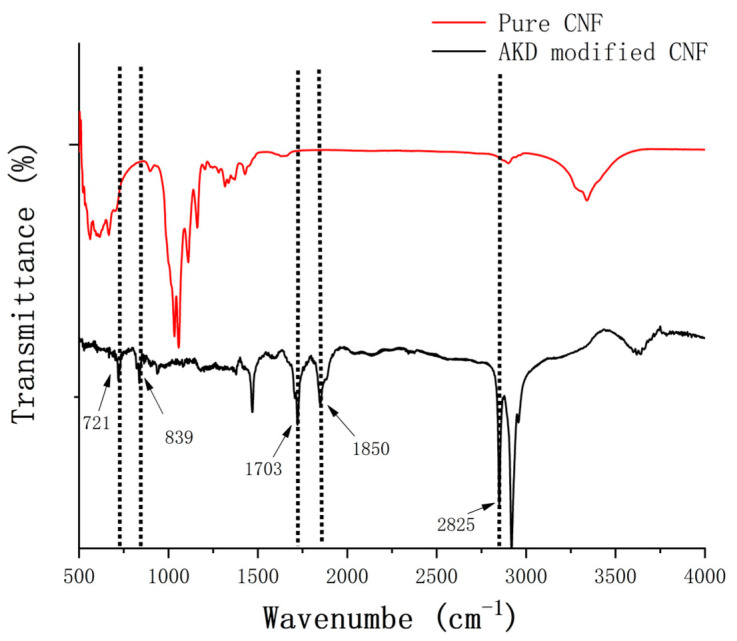
FT-IR spectra of CNF (top) and the AKD modified CNF (bottom).

Figure 3 shows the FTIR spectra of dried CNF and AKD-CNF. From the top spectrum of CNF, it can be observed that one intensive peak appears at 3406 cm^−1^ for the hydrogen bonded —OH stretching, one at 1063 cm^−1^ related to the C—O stretching, and one at 896 cm^−1^ for β-glucosidic bond (the C—H bending and H-C-H stretching). In addition, the characteristic peaks of cellulose are observed at 1376 (the C-H bending), 1645 (the O-H bending) and 2920 cm^−1^ (the C-H stretching) [25]. The FTIR spectrum of AKD-modified CNF was collected (bottom) after sample centrifugation and washed by deionized water in turn for ten times, which presents different peaks largely and indicates the variation of cellulose molecular structure. New peaks appear at 2825, 1850,1703, 839 and 721 cm^−1^, demonstrating the formation of ketone ester structure through the reaction between AKD and the hydroxyl groups of cellulose. The main differences between the two FTIR spectra in Figure 3 are collected in Table 1.

### 3.4. TGA Analysis

**Figure 4:**
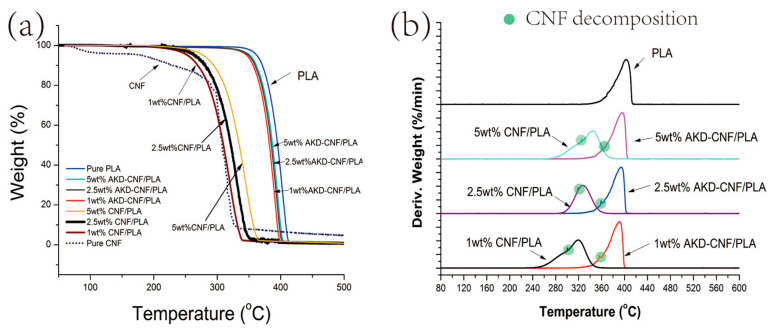
(**a**)TGA thermograms for PLA, its nanocomposites with 1, 2.5 and 5 wt.% AKD-CNF, and with 1, 2.5, 5 wt.% unmodified CNF; (**b**) DTG thermograms for PLA and its nanocomposites with 1, 2.5 and 5 wt.% AKD-CNF, and with 1, 2.5 and 5 wt.% unmodified CNF.

Figure 4 shows the TGA and DTG curves of the CNF, pure PLA and AKD-CNF/PLA. The TGA and DTG curves of unmodified CNF/PLA composites are also collected as control groups and presented in Figure 4. The AKD-CNF/PLA composites exhibit higher thermal stability than the CNF/PLA composites. As shown in Figure 4a, the onset temperatures of the decomposition for pure PLA and AKD-CNF/PLA are almost same at 310 °C. However, the extensional onset temperature of decomposition for PLA and AKD-CNF/PLA are different. The extensional onset temperature of pure PLA is approximately 370 °C in contrast to 350 °C for 1, 2.5 and 5 wt.% AKD-CNF/PLA. The addition of AKD modified CNF does not significantly decrease the thermal stability of PLA and the amount of AKD-CNF hardly affects thermal stability of PLA. Nevertheless, the onset temperature of the decomposition of unmodifed CNF/PLA decreased significantly to approximately 230 °C. The incompatible interfaces between hydrophilic CNF and hydrophobic PLA may attribute to loss of thermal stability although more unmodified CNF addition can improve thermal stability of PLA according to TG curves of CNF/PLA in Figure 4a.

DTG is the first derivative of weight loss. From the DTG curves in Figure 4b, it is observed that the maximum decomposition rate of pure PLA, the peak of DTG curves, is 402.6 °C, in contrast to 391 °C of 1 wt.%, 394 °C of 2.5 wt.% and 396.3 °C of 5 wt.% CNF/PLA. In addition, one little shoulder peak appears in each DTG curve of the three CNF/PLA, respectively, deriving from the CNF decomposition in the nanocomposites. The temperature of the shoulder peak is relatively stable in spite of the change of AKD-CNF content. The variation of AKD-CNF content has little impact on the thermal stability of the composites. As control groups, the maximum decomposition rate of unmodified CNF/PLA composites decreased greatly from 344 °C of 5 wt.% CNF/PLA to 327.6 °C of 2.5 wt.% CNF/PLA and 319 °C of 1 wt.% CNF/PLA. The temperature of the shoulder peak decreased when the content of unmodified CNF reduced, denoting weaker thermal stability of CNF with lower content due to the incompatibility of PLA. All Temperatures at DTG peaks for reinforced PLA composites (Figure 4b) can be found in Table 2.

### 3.5. Morphology of the AKD-CNF/PLA Nanocomposites

**Figure 5:**
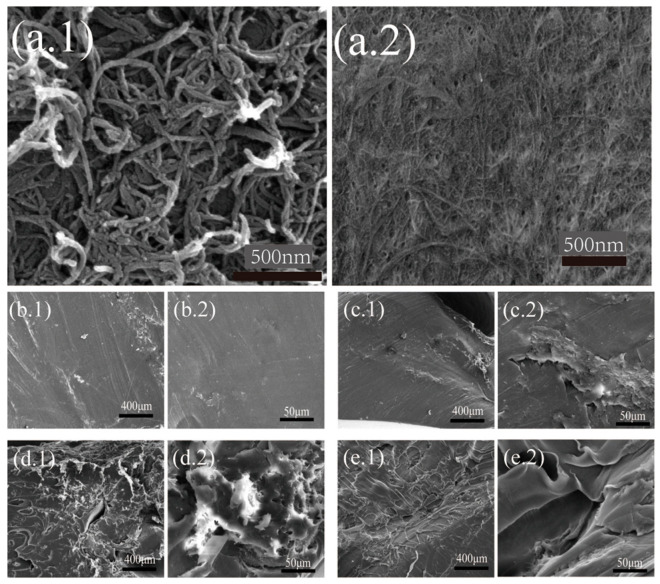
SEM images of (**a**) CNF prepared at (**a.1**) a low stirring speed and (**a.2**) 37,000 rpm; (**b**) pure PLA at (**b.1**) low magnification and (**b.2**) high magnification; (**c**) 1 wt.% AKD-CNF/PLA at (**c.1**) low magnification and (**b.2**) high magnification; (**d**) 2.5 wt.% AKD-CNF/PLA at (**d.1**) low magnification and (**d.2**) high magnification; (**e**) 5 wt.% AKD-CNF/PLA at (**e.1**) low magnification and (**e.2**) high magnification.

Figure 5 shows the FE-SEM images of CNF with various diameters, pure PLA and AKD-CNF/PLA nanocomposites with various contents. Figure 5a presents obtained CNF with various diameters due to different stirring speeds in the high-speed blender. The nanofibers in Figure 5(a.2) were prepared with a stirring speed of 37,000 rpm. All experiments were performed using the same batch of CNFs similar to the nanofiber in Figure 5(a.2), with a constant diameter of approximately 50 nm and the length diameter ratio more than 50. The nanofibers shown in Figure 5(a.1) obviously has a smaller length diameter ratio.

Figure 5b–e presents the morphologies of CNF/PLA composites with different mass ratios measured under different magnifications. It is observed from Figure 5a that pure PLA has a smooth flat surface. When AKD-CNF was added, the surface of PLA turned to much rough. Meanwhile, the level roughness increased along with more AKD-CNF addition.

### 3.6. Mechanical Tests

**Figure 6:**
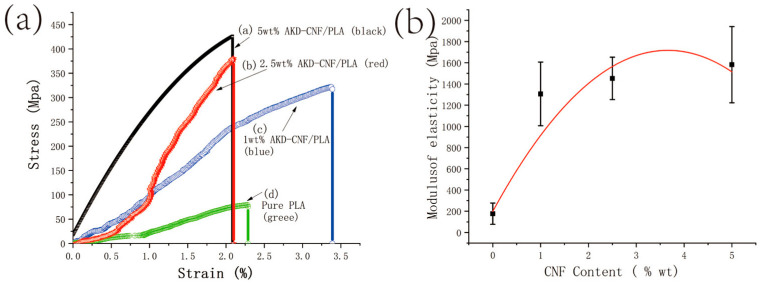
(**a**) Stress–strain curves of the samples of pure PLA (green), 1 wt.% CNF/PLA (blue), 2.5 wt.% CNF/PLA (red) and 5 wt.% CNF/PLA (black) nanocomposites; (**b**) Modulus elasticity-AKD-CNF content relationship curve.

The mechanical properties of the PLA composites reinforced with 1.0, 2.5 and 5.0 wt.% of AKD modified CNF were measured by tensile testing at room temperature. Figure 6a shows the stress–strain curves of pure PLA and AKD-CNF/PLA composites. The curve d in Figure 6a shows the typical elastomeric behavior of pure PLA. The stress increased very slowly when the strain increased largely, indicating susceptible deformation and inferior mechanical properties of pure PLA. The addition of AKD-CNF significantly enhanced the mechanical properties of the composites, as shown by curves a to c in Figure 6a. The data values in Figure 6a are listed in Table 3, including stress at breaking point and ultimate elongation of PLA and AKD-CNF/PLA composites. The ultimate elongation greatly increased from 2.28% of pure PLA to 3.37% of 1 wt.% AKD-CNF/PLA nanocomposite. Although more content of AKD-CNF has a smaller elongation at breaking point. Furthermore, to produce 2.0% deformation, the applied stress increased from 70 MPa for pure PLA to 230 MPa of 1 wt.% CNF/PLA, 380 MPa of 2.5 wt.% CNF/PLA and 427 MPa of 5 wt.% CNF/PLA. The highest values of tensile strength shown in Figure 6a were approximately 2.1% deformation for the AKD-CNF/PLA composite loaded with 5 wt.% of AKD-CNF nanofibers, increasing nearly nine times from the pure PLA. This can be attributed to the compatibility of the interfaces between AKD modified CNF and PLA molecules [30]. Figure 6b presents the effects of AKD-CNF content on the elasticity modulus of PLA. The elasticity modulus showed a significant change once AKD-CNF were added and increased when more AKD-CNF contents are added. The elasticity modulus may reach maximum when the content of AKD-CNF was approximately 5 wt.% according to the curve fitting. Fillers play an important role in determining the mechanical properties of AKD-CNF/PLA composites. The interfacial adhesion between cellulose nanofibers and PLA matrix is one main factor that affect the mechanical properties of CNF reinforced materials. Normally less AKD amount does not alter the cellulose sizing result significantly, so the mechanical properties of the composite mainly determined by the content of CNF.

## 4. Conclusions

PLA composites were prepared from AKD modified cellulose nanofibers (CNF) using DMSO medium. The SEM images show a good dispersion of CNF. The compatible interfaces between CNF and PLA through AKD molecules can be considered as an excellent factor to improve mechanical properties, such as elastic modulus to a level of nine times higher than that of pure PLA when 5 wt.% CNF content is dispersed into the PLA matrix. The morphology of CNF modified PLA turned to rough due to nanofibers incorporated to alter the microstructure of PLA. In addition, thermal properties of AKD-CNF/PLA composites maintained well in comparison with pure PLA although approximately 10 °C was decreased for the extension decomposition temperature. In a word, AKD modified CNF can efficiently improve the mechanical performance of PLA. The present work provides a new idea for the applications of CNF in polymer reinforcements.

## Figures and Tables

**Table 1:** Main Differences of FTIR Signals between CNF and AKD-CNF.

Pure CNF		AKD Modified CNF	
Wavenumber (cm^−1^)	Vibrational Band Assignment	Wavenumber (cm^−1^)	Vibrational Band Assignment
896	β-glucosidic bond	721	cis alkene sp^2^ C-H bend
1063	C-O stretching	839	In-plane bending of C-H
1376	C-H stretching	1703	ν C = O
1645	O-H bending	1850	ν C = O
2920	C-H stretching	2825	ν CH3
3406	Hydrogen bonded O-H stretching		

**Table 2:** Temperatures at DTG Peaks (Figure 4b) for Reinforced PLA composites.

Sample	Temperature at DTG Peaks (°C)	Temperature at CNF Decomposition (°C)
PLA	402.6	N/A
1 wt.% AKD-CNF/PLA	391	356
2.5 wt.% AKD-CNF/PLA	394	359
5 wt.% AKD-CNF/PLA	396.3	361
1 wt.% CNF/PLA	319	300.6
2.5 wt.% CNF/PLA	327.6	318.9
5 wt.% CNF/PLA	344	325

**Table 3:** Mechanical parameters from the stress–strain curves of samples.

	Sample	PLA	1 wt.% AKD-CNF/PLA	2.5 wt.% AKD-CNF/PLA	5 wt.% AKD-CNF/PLA
Parameter					
Stress at breaking point (MPa)		80	321	380	427
Ultimate elongation (%)		2.28	3.37	2.10	2.08

## Data Availability

Data sharing not applicable.
